# eEF2K promotes PD-L1 stabilization through inactivating GSK3β in melanoma

**DOI:** 10.1136/jitc-2021-004026

**Published:** 2022-03-28

**Authors:** Xisha Chen, Kuansong Wang, Shilong Jiang, Hongyin Sun, Xuanling Che, Minghui Zhang, Jiaying He, Ying Wen, Mengting Liao, Xiangling Li, Xiaoming Zhou, Jianxun Song, Xingcong Ren, Wenjun Yi, Jinming Yang, Xiang Chen, Mingzhu Yin, Yan Cheng

**Affiliations:** 1 Department of Pharmacy, The Second Xiangya Hospital, Central South University, Changsha, China; 2 Hunan Provincial Engineering Research Centre of Translational Medicine and Innovative Drug, Changsha, China; 3 Department of Pathology, Xiangya hospital and Department of Pathology, School of Basic Medicine, Central South University, Changsha, China; 4 Department of Dermatology, Hunan Engineering Research Center of Skin Health and Disease, Hunan Key Laboratory of Skin Cancer and Psoriasis, Xiangya Hospital, Central South University, Changsha, China; 5 Department of Medical Oncology, Harbin Medical University Cancer Hospital, Harbin, China; 6 Department of General Surgery, The Second Xiangya Hospital, Central South University, Changsha, China; 7 Department of Pharmacy, School of Medicine, Hunan Normal University, Changsha, China; 8 Department of Microbial Pathogenesis and Immunology, Texas A&M University Health Science Center, Bryan, Texas, USA; 9 Department of Cancer Biology and Toxicology, Department of Pharmacology, College of Medicine, Markey Cancer Center, University of Kentucky, Lexington, Kentucky, USA

**Keywords:** biomarkers, tumor, drug therapy, combination, immunotherapy, melanoma, tumor microenvironment

## Abstract

**Background:**

Immune checkpoint blockade (ICB) targeting programmed death ligand-1 (PD-L1)/programmed cell death protein-1 (PD-1) pathway has become an attractive strategy for cancer treatment; however, unsatisfactory efficacy has limited its clinical benefits. Therefore, a more comprehensive understanding of the regulation of PD-L1 expression is essential for developing more effective cancer immunotherapy. Recent studies have revealed the important roles of eukaryotic elongation factor 2 kinase (eEF2K) in promoting epithelial-mesenchymal transition (EMT), angiogenesis, tumor cell migration and invasion; nevertheless, the exact role of eEF2K in the regulation of tumor immune microenvironment (TIME) remains largely unknown.

**Methods:**

In this study, we used a cohort of 38 patients with melanoma who received anti-PD-1 treatment to explore the association between eEF2K expression and immunotherapy efficacy against melanoma. Immunoprecipitation-mass spectrometry analysis and in vitro assays were used to examine the role and molecular mechanism of eEF2K in regulating PD-L1 expression. We also determined the effects of eEF2K on tumor growth and cytotoxicity of CD8^+^ T cells in TIME in a mouse melanoma model. We further investigated the efficacy of the eEF2K inhibition in combination with anti-PD-1 treatment in vivo.

**Results:**

High eEF2K expression is correlated with better therapeutic response and longer survival in patients with melanoma treated with PD-1 monoclonal antibody (mAb). Moreover, eEF2K protein expression is positively correlated with PD-L1 protein expression. Mechanistically, eEF2K directly bound to and inactivated glycogen synthase kinase 3 beta (GSK3β) by phosphorylating it at serine 9 (S9), leading to PD-L1 protein stabilization and upregulation, and subsequently tumor immune evasion. Knockdown of eEF2K decreased PD-L1 expression and enhanced CD8^+^ T cell activity, thus dramatically attenuating murine B16F10 melanoma growth in vivo. Clinically, p-GSK3β/S9 expression is positively correlated with the expressions of eEF2K and PD-L1, and the response to anti-PD-1 immunotherapy. Furthermore, eEF2K inhibitor, NH125 treatment or eEF2K knockdown enhanced the efficacy of PD-1 mAb therapy in a melanoma mouse model.

**Conclusions:**

Our results suggest that eEF2K may serve as a biomarker for predicting therapeutic response and prognosis in patients receiving anti-PD-1 therapy, reveal a vital role of eEF2K in regulating TIME by controlling PD-L1 expression and provide a potential combination therapeutic strategy of eEF2K inhibition with ICB therapy.

## Introduction

Overexpression of immunoglobulin-like immunosuppressive molecules in cancer cells lead to evasion of immune surveillance and sustain cell survival and tumor progression. With the idea that immune evasion is one of the hallmarks of tumors being proposed and well recognized, the mechanisms underlying tumor-associated immunosuppression and the novel therapeutic strategies have been pursued recent years. The most well studied and understood is T cell-based recognition and destruction of cancer cells, which is accomplished via binding of the T cell receptor on T cells to major histocompatibility complex (MHC) on target cancer cells. Nevertheless, the outcome of this affair is regulated by a range of co-stimulatory and co-inhibitory receptors and their corresponding ligands, which is also known as immune checkpoints. Binding of the programmed death ligand-1 (PD-L1) on cancer cells to its receptor programmed cell death protein-1 (PD-1) on T lymphocytes delivers inhibitory signals, leading to suppression of T lymphocyte proliferation, activation, cytokine release and cytolytic activity.^1 2^ Upregulation of PD-L1 is used by cancer cells to escape T cell-mediated immune surveillance and attacks.^3^ Thus, blockade of the PD-L1/PD-1 axis has been considered as a promising therapeutic strategy and shows encouraging clinical outcomes in melanoma and other cancers.^3–6^ Previous studies have revealed several proteins that play important roles in the post-translational regulation of PD-L1 stability, which include OTUB1, CSN5, glycogen synthase kinase 3 beta (GSK3β), CDK4/CDK6, CMTM4/6, palmitoylated B3GNT3, SPOP (speckle-type POZ protein) and β-TrCP (beta-transducin repeats-containing proteins) were identified.^7–12^ Further understanding of the molecule mechanisms underlying PD-L1 regulation will help identify biomarkers and develop new strategies to overcome limitations and improve efficacy of current PD-L1/PD-1 blockade therapies.

Eukaryotic elongation factor 2 kinase (eEF2K), a calcium/calmodulin-activated member of the α-kinase family, inhibits messenger RNA (mRNA) translation elongation by phosphorylating and inactivating its substrate eEF2, which mediates the movement of polypeptidyl-tRNAs from the A site to the P site of the ribosome.^13–15^ eEF2K is overexpressed in a variety of malignancies including pancreatic, brain and breast cancer, and promotes cell survival under conditions of nutrient deprivation, hypoxia and therapeutic stress.^16–18^ Our previous study found that eEF2K promotes cancer cell proliferation by regulating aerobic glycolysis and blockade of glycolysis by targeting eEF2K could inhibit tumor development and enhance sensitivity of breast cancer cells to chemotherapy drugs.^19^ In addition, we demonstrated that eEF2K is an important autophagic modulator and plays a critical regulatory role in drug-induced autophagy in cancer cells.^20–22^ Therapeutic targeting of eEF2K increased drug efficacy by inhibiting autophagy.^23^ Recently, several studies revealed the important roles of eEF2K in promoting EMT, angiogenesis, tumor cell migration and invasion.^24–27^ However, the exact role of eEF2K in the regulation of tumor immune microenvironment remains largely unknown.

In this study, we found that higher eEF2K expression correlates with a better therapeutic outcome and a prolonged survival in patients with melanoma treated with anti-PD-1 immunotherapy. More importantly, there is a positive correlation between eEF2K and PD-L1 expression. We further showed that eEF2K promotes stabilization of PD-L1 protein through its interaction with GSK3β, and that knockdown of eEF2K decreased PD-L1 expression and increased CD8^+^ T cell number and granzyme B (GZMB) in tumor tissues in a mouse melanoma model. Moreover, we characterized the enhanced efficacy of eEF2K knockdown or NH125, an eEF2K inhibitor, in combination with anti-PD-1 in vivo. Our results reveal a critical role of eEF2K in regulating tumor immune microenvironment by controlling PD-L1 expression, and provide a new approach to enhancing cancer immunotherapy by targeting eEF2K.

## Materials and methods

### Cell lines and culture

The human melanoma cell lines, A375, SK-5 and SK-28 were cultured in Dulbecco’s Modified Eagle Medium. The murine B16F10 cells were maintained in RPMI 1640. All cell culture media were supplemented with 10% fetal bovine serum, penicillin (100 U/mL) and streptomycin (100 μg/mL). All cell lines were maintained at 37°C in a humidified atmosphere containing 5% CO_2_/95% air and used within 3–20 passages of thawing the original stocks.

### Reagents and antibodies

The proteasome inhibitor MG132 and protein synthesis inhibitor cycloheximide (CHX) were purchased from Selleck. Antibodies used in immunoblotting: eEF2K (ab45168, ab85721, 1:1000) was purchased from Abcam; PD-L1 (17952-1-AP, 1:1000), GSK3β (22104-1-AP, 1:2000), β-actin (60008-1-lg, 1:5000), Flag (66008-3-lg, 1:5000), tubulin (11224-1-AP, 1:5000), GST (10000-0-AP, 1:4000) were purchased from Proteintech; HA (No. 3724, 1:1000), p-GSK3β/S9 (No. 9323, 1:1000) were purchased from Cell Signaling Technologies; phosphoserine monoclonal antibody (mAb) (CSB-MA080235, 1:1000) was purchased from CUSABIO. Normal IgG/Peroxidase-conjugated AffiniPure Goat Anti-Rabbit/Mouse IgG (H+L) was purchased from Jackson Immuno Research.

### siRNA, short hairpin RNA and plasmid transfection

Transfection of siRNA was carried out according to the manufacturer’s protocol. Briefly, cells in exponential phase of growth were plated in 6-well tissue culture plates at 1×10^5^ cells per well, grown for 24 hours, and then transfected with siRNA using lipofectamine RNAimax reagent and Opti-MEM-reduced serum medium. For the generation of stable cells, the lentiviral-based short hairpin RNA (shRNA) was used to knock down expression of indicated genes. After infection with the corresponding gene-targeted shRNA lentiviral particles for 24 hours, cells stably expressing the shRNA were then selected with 1 μg/mL puromycin. Transfection of the plasmid was carried out using lipofectamine 2000 (Invitrogen) reagent according to the manufacturer’s protocol.

### Western blot analysis and immunoprecipitation

Western blot analysis was performed as described previously.^28^ For immunoprecipitation, the cells were lysed with RIPA lysis buffer (Medium) supplemented with protease inhibitors and/or phosphatase inhibitors at ice for 30 min, followed by centrifugation at 16,000 g for 15 min to remove debris. Cleared lysates were then subjected to immunoprecipitation with indicated primary antibody and protein A/G agarose beads (from Santa Cruz) at 4°C overnight. The immunocomplexes were then washed four times with RIPA buffer the next day, and proteins were boiled in sodium dodecyl sulfate-polyacrylamide gel electrophoresis (SDS-PAGE) sample buffer for 10 min, followed by western blot analysis.

### Pulse-chase assay

To measure the effect of eEF2K on PD-L1 protein stability, the A375 cells transfected with the indicated siRNAs were treated with the protein synthesis inhibitor CHX (50 µg/mL) for the indicated durations before collection, and then subjected to western blot analysis.

### Detection of cell surface PD-L1

For detection of cell surface PD-L1, cells were suspended in 100 µL of cell staining buffer and incubated with APC-conjugated antihuman PD-L1 antibody (APC-65081, Proteintech) at 4°C for 30 min. After washing in the staining buffer, stained cells were analyzed by fluorescence activated cell sorting (FACS).

### GST pulldown assay

Purified GST or GST-eEF2K from bacteria bound to glutathione-sepharose 4B beads (from sigma) was incubated with GSK3β for 4 hours at 4°C. The beads were then washed with GST binding buffer four times, and the bound proteins were separated by SDS-PAGE and immunoblotted with indicated antibodies.

### Immunofluorescence staining

A375 cells seeded on glass coverslip were fixed in 4% paraformaldehyde for 20 min at room temperature and blocked in 5% bovine serum albumin (BSA) for 1 hour. Then, cells were incubated with anti-eEF2K antibody (Abcam, ab96685, 1:50) and anti-GSK3β antibody (Proteintech, 67329-1-lg, 1:50) at 4°C for overnight, followed by Alexa Fluor 594 dye-conjugated antimouse IgG antibody and Alexa Fluor 488 dye-conjugated antirabbit IgG antibody. At the end of incubation, the cells were stained with 4,6-diamidino-2-phenylindole (DAPI). The coverslip was washed in phosphate-buffered saline (PBS) and fluorescent signals were visualized using a confocal microscope.

For tumor tissue immunofluorescence (IF), 4 μm paraffin sections of tissue samples were baked for 120 min at 60°C, and then deparaffinized. Antigen was retrieved at citric acid buffer by microwave antigen retrieval, followed with 3% BSA blocking for 1 hour at room temperature. Primary antibodies were incubated at 4°C for overnight and the following day for 30 min at room temperature. After washing with TBS-0.25% Triton X-100, the secondary antibody was added to the blocking solution and incubated for 1 hour. At the end of incubation, tissue sections were incubated with DAPI solution at room temperature for 5 min and images were detected and captured by confocal microscope.

### Patients and tissue samples

A total of 38 patients with acral melanoma with standardized PD-1 mAb therapy from the research files at The Tumor Hospital of Harbin Medical University who were seen from January 2016 to December 2020 (PD-1 mAb, Keytruda, Merck KGaA, Germany), and who met our inclusion criteria, were entered in this study. The eligibility criteria included the following: (1) pathological examination confirming the presence of stage Ⅲ–Ⅳ melanoma; (2) complete basic clinical data; (3) no serious complications or other malignant disease; (4) the patients and family members being informed about the illness and having given informed consent before treatment.

### Immunohistochemical staining

Paraffin-embedded implantation samples isolated from peritoneal metastasis of patients with acral melanoma (38 cases) were sectioned at a thickness of 4 µm. To stain eEF2K, p-GSK3β and PD-L1, the slides were first deparaffinized in xylene and rehydrated with gradient concentrations of alcohol under standard procedures. After rehydration, the slides were immersed in 0.01 mol/L citrate buffer (pH 6.0) and heated (95°C) for 15 min for antigen retrieval. Then, the samples were incubated with 3% hydrogen peroxide (H_2_O_2_) for 10 min followed by 10% normal goat serum blocking for 10 min. Subsequently, the sections were incubated with rabbit polyclonal antihuman eEF2K antibody (dilution 1:100) (ab85721, Abcam) and antihuman p-GSK3β (dilution 1:50) (WL03683, Wanleibio), or with mouse monoclonal antihuman PD-L1 (dilution 1:100) (17952-1-AP, Proteintech) for 1 hour at room temperature. After washing with PBS with Tween 20 for three times, the sections were incubated with biotin-labeled secondary antibody followed by horseradish peroxidase (HRP)-conjugated streptavidin for 30 min individually at room temperature. After applying HRP substrate, 3.3′-diaminobenzidine tetrahydrochloride (D3939-1set, Sigma) in 0.01% H_2_O_2_, for 10 min, the slides were counterstained with Meyer’s hematoxylin for 30–60 s and mounted with mounting medium for visualization under microscope.^29 30^ Scoring of eEF2K, p-GSK3β and PD-L1 in melanoma samples via immunohistochemical (IHC) staining follows the methods previously published. All of IHC staining samples from patients with melanoma were evaluated independently by two experienced pathologists.

### Semi-quantitative analysis of IHC staining

All of the samples were reviewed by two independent pathologists experienced in evaluating IHC, who were blinded to the clinical outcome of these patients. We assessed the percentage of positively stained immunoreactive cells and the staining intensity to semi-quantitatively determine the expression of eEF2K, PD-L1, p-GSK3β. The percentage of immunoreactive cells was rated as follows: 0 points, <10%; 1 point, 10%–50%; 2 points, >50%. The staining intensity was rated as follows: 0 (no staining or weak staining=light yellow), 1 (moderate staining=yellow brown) and 2 (strong staining=brown). The overall score for eEF2K, PD-L1, p-GSK3β expression was the sum of points determined for the percentage of positively stained immunoreactive cells and the expression, and an overall score ranging from 0 to 4 was assigned. For the statistical analysis, the patients were divided into a low expression group (an overall score between 0 and 2) and a high expression group (an overall score between 3 and 4).^29–31^ The final score is the combination of independent scores assigned by the two pathologists, which was reported in this study. Any differences in the scores were resolved by discussion between the two pathologists.

### Clinical response and follow-up evaluation

Clinical response was evaluated using the RECIST criteria for solid tumors (V.1.1).^32 33^ Complete response (CR) was defined as complete disappearance of all lesions; partial response (PR) was defined as at least a 30% decrease in the sum of the largest diameter (LD) of the targeted lesions; stable disease (SD) was defined as neither shrinkage that qualified as PR nor sufficient increase that qualified as progressive disease (PD) and PD was defined as at least a 20% increase in the sum of the LD of the target lesions. Response was defined as CR plus PR and non-response was defined as SD plus PD. The clinical response was evaluated based on imaging only. The images were evaluated by two experienced radiologists aware of patient diagnosis and treatment but not the results of other imaging modalities. On discrepancy between the two readings, a third, independent experienced radiologist served as the final arbitrator.

During the follow-up period, patients with melanoma with post-PD-1 antibody treatment were requested to perform CT scan of the lungs and color Doppler ultrasound of the liver and kidney every 3 months in the first 2 years, every 6 months from 3 to 5 years and annually thereafter. The outcome of melanoma was defined as good prognosis (improvement or full resolution) or bad prognosis (no recovery or death due to melanoma). The end points of the study were progression-free and overall survival (OS) as reported.

### In vitro kinase assay

For in vitro kinase assay, 1 µg recombinant GSK3β was incubated with 200 ng active eEF2K in 1× kinase buffer (Cell Signaling Technology, 9802) supplemented with ATP (Cell Signaling Technology, 9804) at a final concentration of 200 μM. After incubation for 1 hour at 30°C, the reaction was terminated by the addition of SDS-PAGE sample loading buffer and followed by western blot analysis.

### PD-L1 and PD-1 interaction assay

To measure the binding of PD-1 to PD-L1, A375 cells transfected with siNT and sieEF2K were seeded into 6-well cell culture plates and then fixed in 4% paraformaldehyde at room temperature for 15 min, followed by incubation with recombinant human PD-1 Fc chimera protein (MCE) for 1 hour. After washing with phosphate-buffered saline, cells were incubated with antihuman IgG/Alexa Fluor 488 dye conjugate (Bioss) at room temperature for 1 hour, and then Nuclei were stained with DAPI. The green fluorescence signal of Alexa Fluor 488 dye was visualized using a confocal microscope.

### Animal studies

Briefly, B16F10 cells (5×10^5^) were injected subcutaneously into male nude mice and C57BL/6 mice in a volume of 100 μL. Tumor sizes and body weights were measured on the days as indicated and tumor volume was calculated as length×width^2^× (π/ 6). At the termination of the experiment, subcutaneous tumors were excised and weighed, then photopraphs were taken. Tumor tissues were subjected to subsequent further analysis.

For treatment with antibody, 75 μg anti-PD-1 monoantibody (Clone J43, BE0033-2, Bio X cell, West Lebanon, New Hampshire), anti-CD8α (BP0117) or isotype IgG control (BE0091, BP0090) was injected intraperitoneally on days 6, 9, 12, 15 and 18 after B16F10 cell inoculation. For small molecular inhibitor treatment, 500 μg/kg NH125 was injected intraperitoneally every day for 2 weeks.

### Flow cytometry analysis

In this study, all flow cytometry antibodies and agents were purchased from eBiosciences. For mouse samples, B16f10-xenograft tumors were harvested after experiments and then subjected to rapid and gentle stripping, physical grinding and filter filtration to obtain single cell suspension. After getting rid of dead cells with eBioscience Fixable Viability Dye eFluor 780 (65-0865-14) staining, cells were stained with FITC-CD45 (11-0451-81), PerCP/Cy5.5-CD3 (45-0031-80), PE/Cy7-CD8 (25-0081-81) for 30 min. After fixation and permeabilization, intracellular GZMB was stained using APC-GZMB (17-8898-80). Stained samples were analyzed by FACS, and FlowJo software was used for further data analysis.

### Statistical analysis

All experiments were performed at least three times. The results are shown as mean±SD. Comparison between two groups was analyzed using the Student’s t-test. Comparison of multiple groups (>2) were performed using one-way analysis of variance. For the analysis of associations between eEF2K, PD-L1 and p-GSK3β/S9 expression levels, the IF stains were scored, and the correlation analyses were performed using Spearman’s correlation test. Wilcoxon rank sum test was used for the analysis of associations between the response to PD-1 mAb therapy and the indicated protein expression. The Kaplan-Meier method and Gehan-Breslow-Wilcoxon test were used for the survival data analysis. GraphPad Prism V.6.01 was used to perform statistical analysis. P values <0.05 were considered statistically significant.

## Results

### eEF2K expression positively correlates with immunotherapeutic benefits and PD-L1 level in patients with melanoma

**Figure 1:**
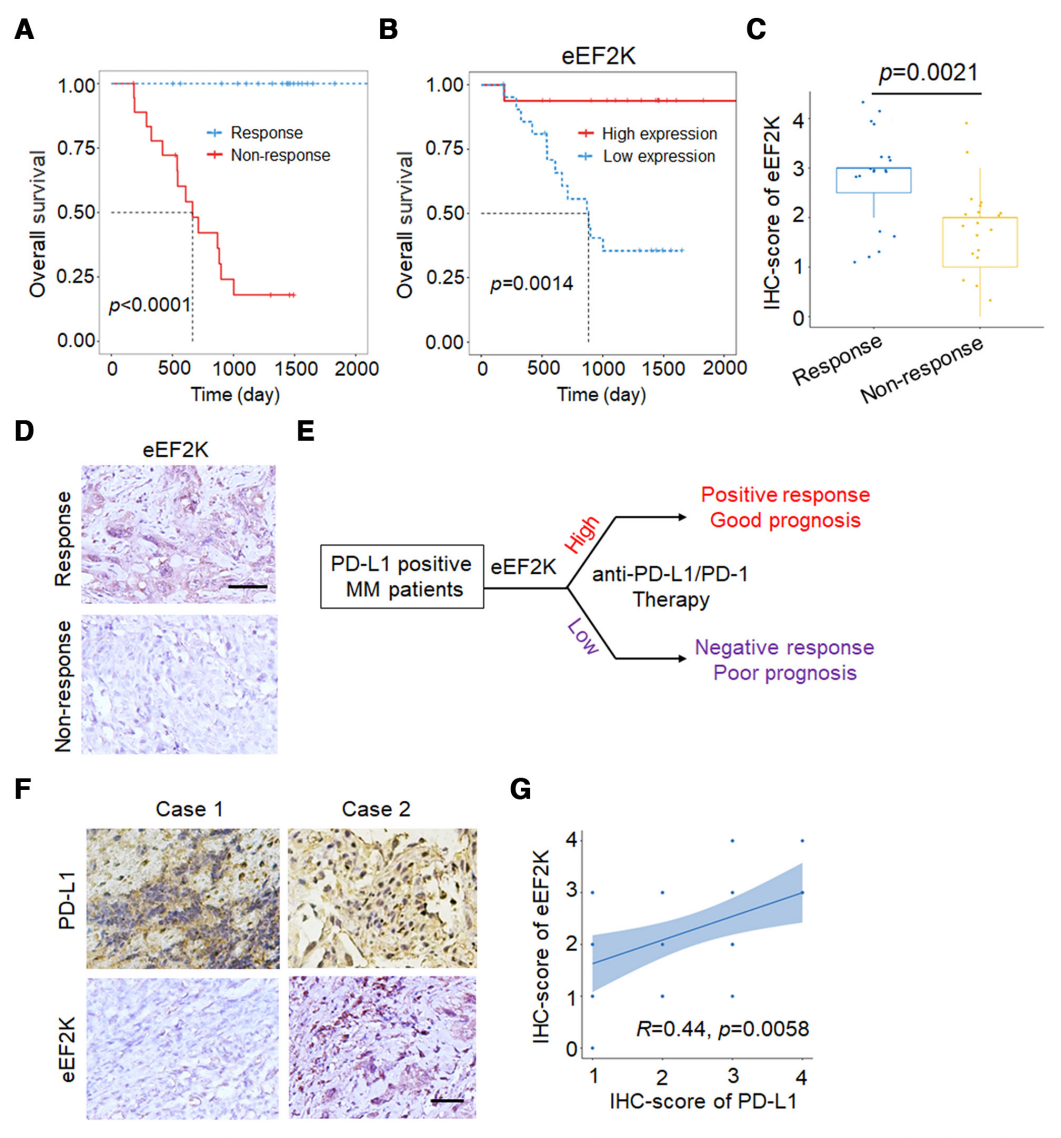
High eEF2K expression positively correlates with immunotherapeutic benefits and PD-L1 level in patients with melanoma. (A) Kaplan-Meier overall survival curves in patients with melanoma with or without response to PD-1 mAb therapy. (B) Kaplan-Meier plots of the overall survival rates in PD-1 blockade-treated patients with melanoma with high (IHC overall score is between 3 and 4) or low (IHC overall score is between 0 and 2) expression of eEF2K. (C) IHC staining of 38 human melanoma specimens was performed with the eEF2K antibody. (D) Representative IHC staining images of eEF2K from respond group and non-respond group are shown, scale bar is 50 μm. (E) Schematic diagram for eEF2K as a predictor for the efficacy and prognosis of anti-PD-L1/PD-1 therapy (good prognosis means improvement or full resolution, poor prognosis means no recovery or death due to melanoma). (F, G) The positive correlation between eEF2K and PD-L1 expression in human melanoma specimens. (F) The IHC staining images, and the correlation analyses were performed (G). eEF2K, eukaryotic elongation factor 2 kinase; IHC, immunohistochemical; mAb, monoclonal antibody; PD-1, programmed cell death protein-1; PD-L1, programmed death ligand-1.

In a cohort of 38 patients with melanoma treated with PD-1 mAb therapy, patients with a positive response exhibited longer survival (figure 1A). We next investigated the role and clinical value of eEF2K in patients with melanoma treated with anti-PD-1 therapy. As shown in figure 1B, patients with high eEF2K levels demonstrated longer OS. Likewise, patients with a positive response to immunotherapy showed higher IHC staining scores of eEF2K (figure 1C, D). All of these results suggest that in patients with positive PD-L1 expression, eEF2K may serve as a potential biomarker to predict the clinical efficacy and prognosis of immunotherapy (figure 1E). Furthermore, the expression of PD-L1 was positively correlated with eEF2K level in tumor samples from patients with melanoma (figure 1F, G). These findings suggest the involvement of eEF2K in the regulation of tumor immune microenvironment, and its usefulness in predicting outcome of immunotherapy in patients with melanoma.

### eEF2K upregulates PD-L1 expression by inhibiting its proteasome-mediated degradation

**Figure 2:**
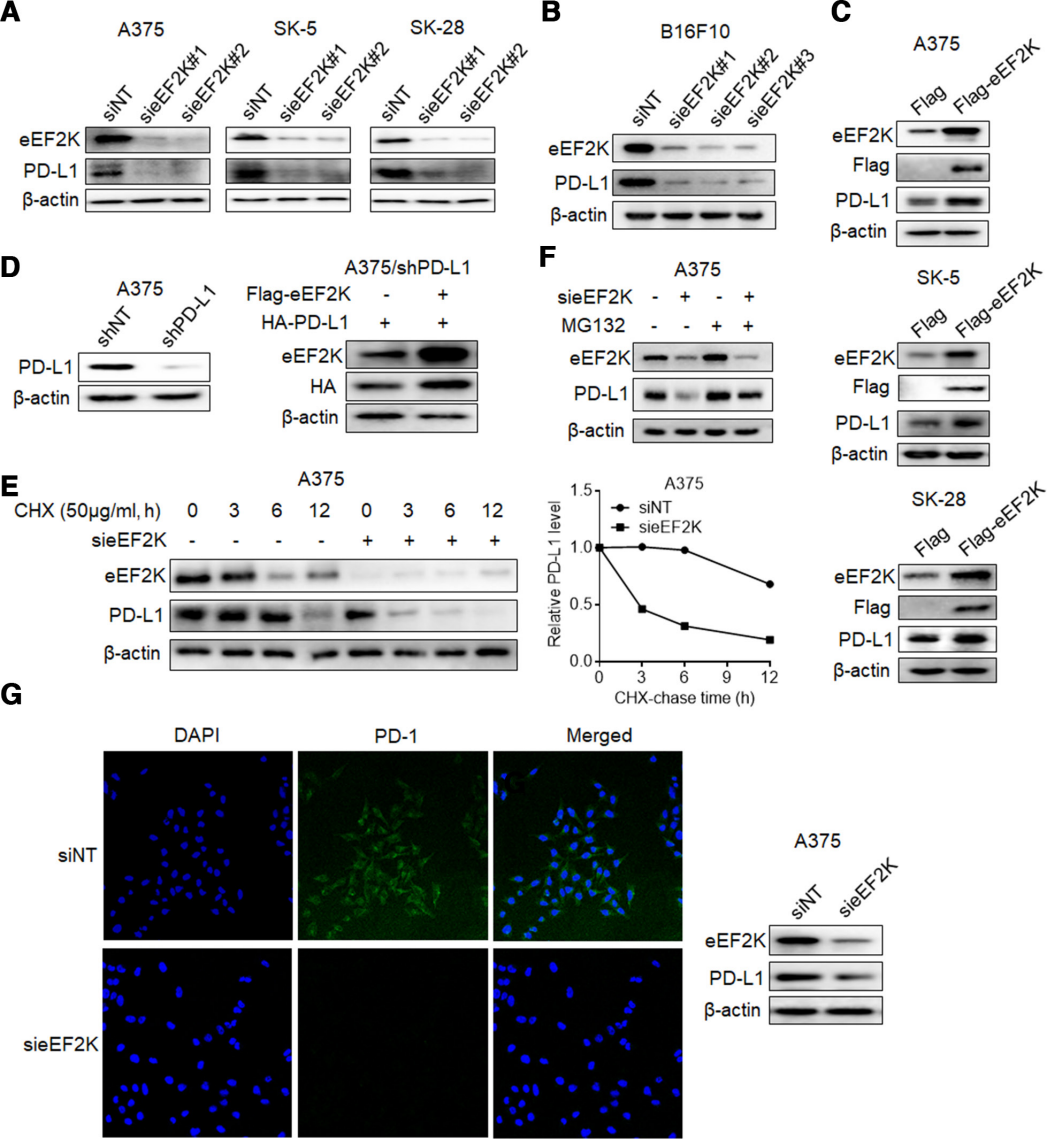
eEF2K upregulates PD-L1 expression by inhibiting its proteasome-mediated degradation. Immunoblotting analyses were performed with the indicated antibodies (A–G). (A) Human melanoma cell lines were transfected with a non-targeting siRNA or homo sapiens eEF2K-targeted siRNAs for 72 hours. (B) B16F10 cells were transfected with a non-targeting siRNA or mouse eEF2K-targeted siRNAs for 72 hours. (C) A375, SK-5, SK-28 cells were transfected with a control plasmid or a Flag-eEF2K plasmid for 48 hours. (D) PD-L1-depleted A375 cells with reconstituted expression of HA-PD-L1 were transfected with a control plasmid or a Flag-eEF2K plasmid for 48 hours. (E) A375 cells with or without eEF2K knockdown were treated with CHX (50 µg/mL) for the indicated times. (F) A375 cells with or without eEF2K knockdown were treated with or without MG132 (10 µM) for 4 hours. (G) A375 cells with or without eEF2K knockdown were incubated with recombinant human PD-1 Fc protein and then antihuman Alexa Fluor 488 dye. Immunofluorescence assays were performed to detect PD-1 binding intensity. CHX, cycloheximide; eEF2K, eukaryotic elongation factor 2 kinase; IHC, immunohistochemical; mAb, monoclonal antibody; PD-1, programmed cell death protein-1; PD-L1, programmed death ligand-1; siRNA, small interfering RNA.

In view of the significant positive correlation between PD-L1 and eEF2K, we evaluated the effect of eEF2K on PD-L1. As shown in figure 2A and online supplemental figure S1A, silencing of eEF2K by two different siRNAs significantly decreased PD-L1 expression in three human melanoma cell lines, and this effect was also shown in the murine melanoma cell line B16F10 (figure 2B, online supplemental figure S1B). By contrast, forced expression of eEF2K upregulated the levels of PD-L1 (figure 2C, online supplemental figure S1C), indicating that eEF2K plays a role in promoting PD-L1 expression. Furthermore, eEF2K silencing induced a significant decrease of cell surface PD-L1 levels (online supplemental figure S1D). However, neither knockdown nor overexpression of eEF2K leaded to obvious change in PD-L1 mRNA levels (online supplemental figure S1E–G). In addition, A375 cells with endogenous PD-L1 expression knocked down were transfected with the coding sequence of PD-L1, and a robust promotion of eEF2K in the ectopically expressed PD-L1 was also observed (figure 2D), implying that PD-L1 is regulated by eEF2K at post-transcriptional level.

10.1136/jitc-2021-004026.supp1 Supplementary data

Next, we investigated the effect of eEF2K on PD-L1 stability by addition of CHX to block new protein synthesis. As shown in figure 2E and online supplemental figure S1H, knockdown of eEF2K dramatically promoted PD-L1 turnover and shortened the half-life of PD-L1. Furthermore, proteasome inhibitor MG132 reversed the eEF2K inhibition-induced PD-L1 downregulation (figure 2F). These results indicate that eEF2K promotes the stabilization of PD-L1 by inhibiting proteasome-mediated degradation.

PD-L1 is known to suppress antitumor immune response via binding to its receptor PD-1.^34^ As eEF2K stabilizes PD-L1, we tested whether eEF2K regulates PD-L1/PD-1 interaction. IF assays revealed that knockdown of eEF2K decreased PD-1 protein binding intensity to the tumor cell surface (figure 2G).

### Stabilization of PD-L1 by eEF2K is mediated by binding to and inactivating GSK3β by phosphorylation

**Figure 3:**
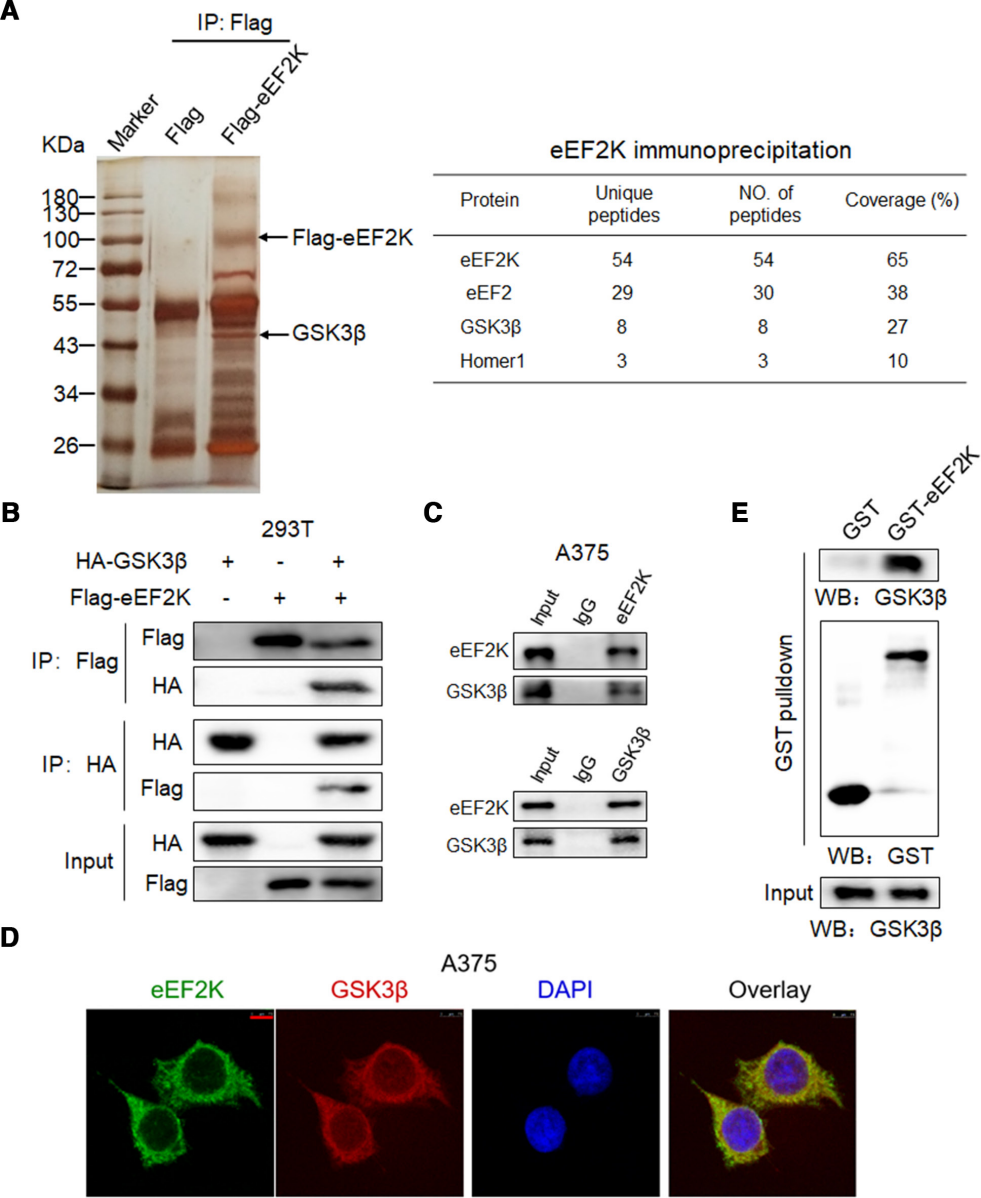
Physical interaction of eEF2K with GSK3β. (A) Proteins from the immunoprecipitates assay were separated by polyacrylamide gel eletrophoresis and detected by sliver staining (left). The proteins immunoprecipitated by anti-Flag antibody were further analyzed by mass spectrometry, and several reported eEF2K-interacting proteins like eEF2 and Homer1 were also identified in our Immunoprecipitation-mass spectrometry (IP-MS) analysis (right). Immunoblotting analyses were performed with the indicated antibodies (B, C and E). (B) HEK293T cells were transfected with Flag-eEF2K and HA-GSK3β plasmids, and then subjected to immunoprecipitation with anti-Flag or anti-HA antibodies. The lysates and immunoprecipitates were then blotted. (C) Immunoprecipitation analysis with the indicated antibodies was performed to detect endogenous eEF2K and GSK3β interaction in A375 cells. (D) eEF2K and GSK3β are co-localized in the cytoplasm of A375 cells. The cellular location of eEF2K and GSK3β was examined by immunofluorescence staining. DAPI was used to stain the DNA. Scale bar, 7.5 μm. (E) Purified recombinant GST-eEF2K interacts with GSK3β. GST-eEF2K and GST proteins were pulled down with glutathione beads. GSK3β was detected by WB. DAPI, 4,6-diamidino-2-phenylindole; eEF2K, eukaryotic elongation factor 2 kinase; GSK3β, glycogen synthase kinase 3 beta.

Since phosphorylation-dependent proteasomal degradation of PD-L1 has been reported, there is a possibility that eEF2K phosphorylates PD-L1 and facilitates its degradation. To verify this hypothesis, we performed immunoprecipitation and western blot analysis to investigate the interaction between eEF2K and PD-L1. As shown in online supplemental figure S2, eEF2K was not detected in the immunoprecipitated cell lysates with antibody against HA, which rules out the above assumption. Then, we performed immunoprecipitation followed by mass spectrometry to identify eEF2K-interacting proteins. In addition to several reported eEF2K-interacting proteins like eEF2^35^ and Homer1,^36^ GSK3β, a key regulator of tumor immunity by inducing phosphorylation-dependent PD-L1 proteasomal degradation,^11^ was shown to be a potential partner of eEF2K (figure 3A). To confirm this result, a co-immunoprecipitation was performed in 293 T cells, and western blot analysis showed that eEF2K associated with GSK3β (figure 3B). Furthermore, we demonstrated the association of endogenous eEF2K and GSK3β in A375 cells (figure 3C). In addition, IF exhibited apparent co-localization of eEF2K and GSK3β (figure 3D). Furthermore, the direct interaction between eEF2K and GSK3β was demonstrated by in vitro GST pulldown assay. As shown in figure 3E, when purified recombinant GST-eEF2K was pulled down by glutathione beads, GSK3β was detected in the complex, suggesting a direct interaction between eEF2K and GSK3β.

**Figure 4:**
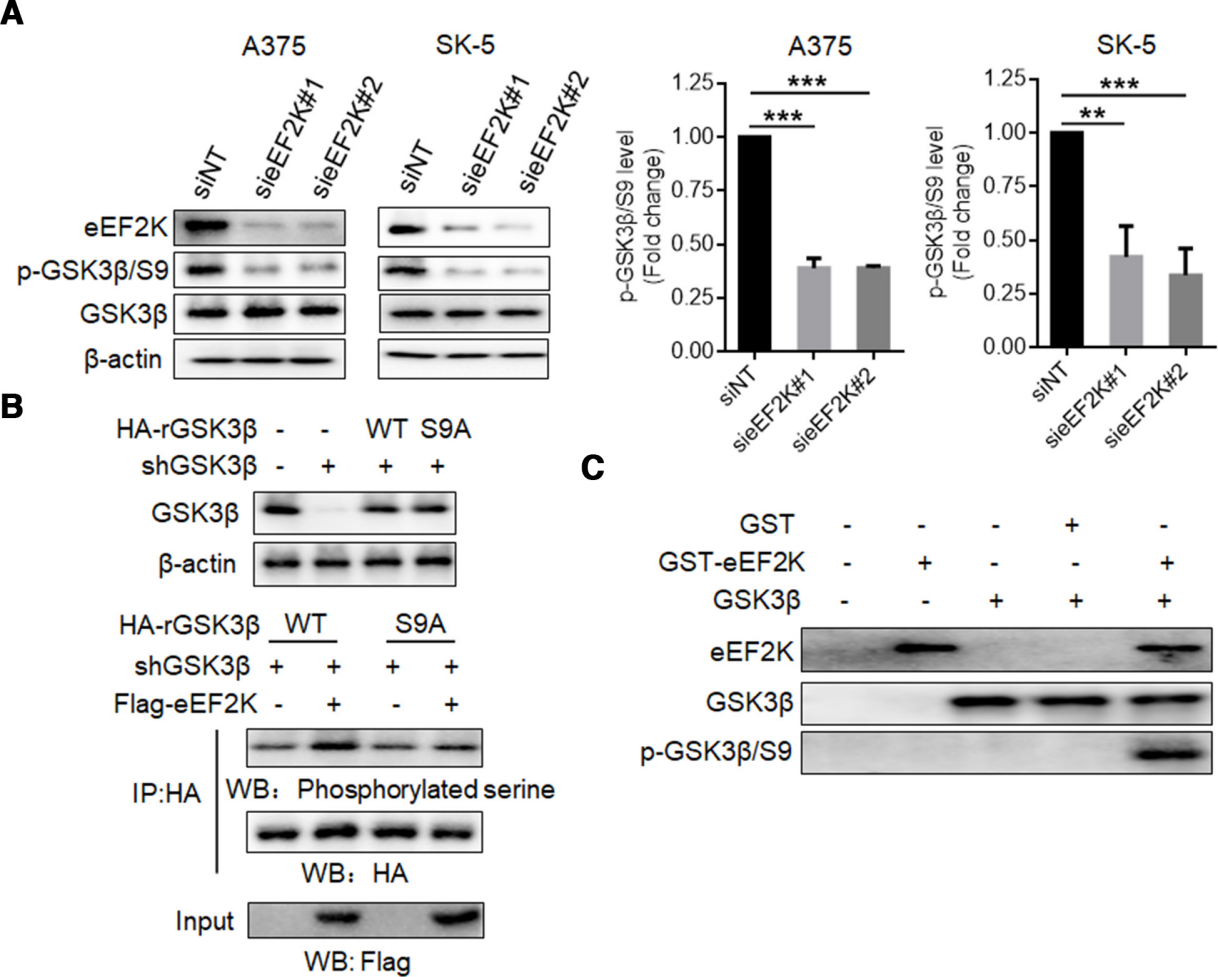
eEF2K phosphorylates GSK3β at serine 9. Immunoblotting analyses were performed with the indicated antibodies. (A) A375 or SK-5 cells were transfected with a non-targeting siRNA or eEF2K-targeted siRNAs for 72 hours. ∗∗P<0.01; ∗∗∗p<0.001. (B) A375 cells with or without GSK3β depletion and reconstituted expression of wild-type (WT) HA-rGSK3β or HA-rGSK3β S9A mutant were transfected with a control plasmid or a Flag-eEF2K plasmid for 48 hours. Immunoprecipitation analysis was performed. (C) Purified GSK3β was incubated with active eEF2K kinase. Western blot analysis was performed with the p-GSK3β/S9 antibody. eEF2K, eukaryotic elongation factor 2 kinase; GSK3β, glycogen synthase kinase 3 beta; siRNA, small interfering RNA.

**Figure 5:**
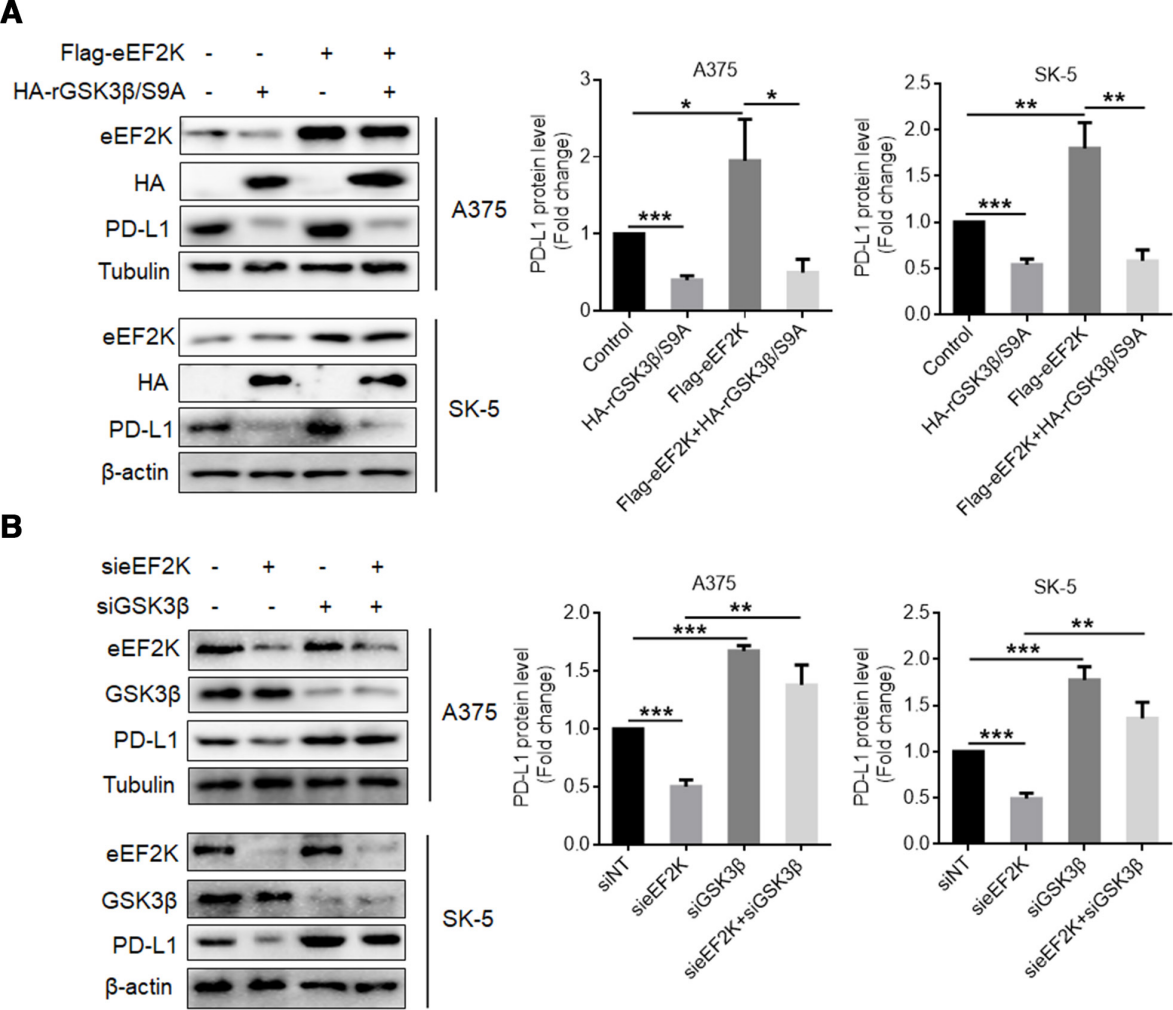
eEF2K-mediated GSK3β inactivation promotes the stabilization of PD-L1. Immunoblotting analyses were performed with the indicated antibodies. (A) Flag or Flag-eEF2K-expressing A375 or SK-5 cells were transfected with or without HA-rGSK3β S9A for 48 hours. (B) A375 or SK-5 cells with or without eEF2K knockdown were transfected with a non-targeting siRNA or GSK3β-targeted siRNA for 72 hours. ∗P<0.05; ∗∗p<0.01; ∗∗∗p<0.001. eEF2K, eukaryotic elongation factor 2 kinase; GSK3β, glycogen synthase kinase 3 beta; PD-L1, programmed death ligand-1; siRNA, small interfering RNA.

As a potential partner of eEF2K, we further analyzed whether eEF2K affects GSK3β phosphorylation in melanoma cells. Figure 4A shows that silencing of eEF2K expression markedly decreased phosphorylation of GSK3β at serine 9 (S9), which has been reported to result in its inactivation.^37^ To further clarify the role of this residue in the effect of eEF2K on GSK3β phosphorylation, we silenced endogenous GSK3β and transfected small interfering RNA (siRNA)-resistant-HA-GSK3β (HA-rGSK3β) plasmid or siRNA-resistant-HA-GSK3β S9A mutant plasmid in 293 T cells. As shown in figure 4B, eEF2K overexpression increased serine phosphorylation of WT HA-rGSK3β but not HA-rGSK3β S9A mutant, indicating that S9 is essential for eEF2K-mediated phosphorylation of GSK3β. Furthermore, in vitro kinase assays also revealed that eEF2K directly phosphorylates GSK3β at S9 (figure 4C). These results prove that eEF2K interacts with and inactivates GSK3β by phosphorylating it at S9. We further determined whether GSK3β truly mediates the stabilizing effect of eEF2K on PD-L1. HA- rGSK3β S9A (constitutively active) was transfected into A375 or SK-5 cells with or without eEF2K overexpression, and the expression of PD-L1 was measured. The results showed that overexpression of the GSK3β mutant reversed eEF2K-induced PD-L1 upregulation (figure 5A). Inversely, silencing of GSK3β rescued PD-L1 reduction caused by eEF2K knockdown (figure 5B). As GSK3β-induced PD-L1 phosphorylation facilitates its degradation and downregulation, we found the increased PD-L1 phosphorylation in eEF2K knockdown cells (online supplemental figure S3). These observations support that eEF2K-phosphorylated GSK3β enhanced PD-L1 stability.

### Depletion of eEF2K suppresses tumor growth and PD-L1 expression in vivo

**Figure 6:**
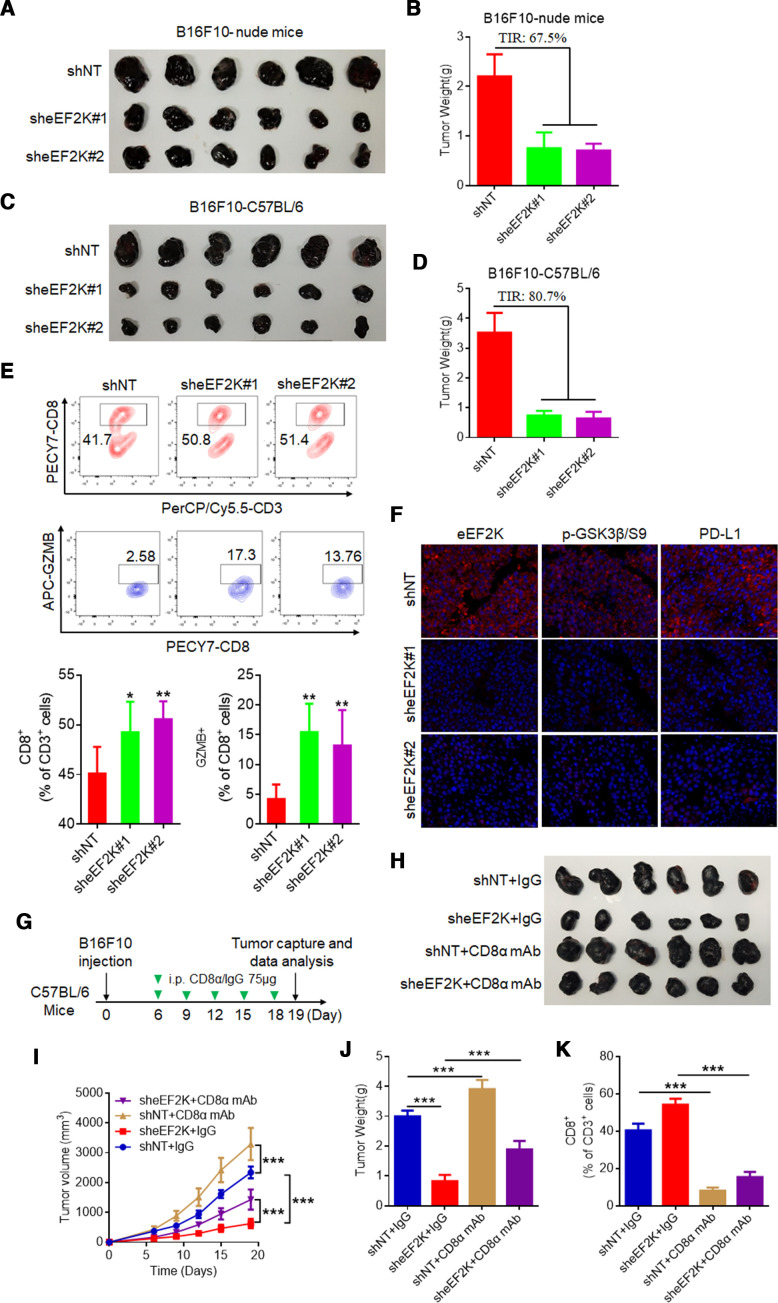
Depletion of eEF2K suppresses B16F10 xenograft tumor growth and promotes T cell activity. Ctrl and two sheEF2K (#1 and #2)-transfected B16F10 cells were injected subcutaneously into 6-week-old male Balb/c nude mice and C57BL/6 mice, the tumor sizes were measured on the days as indicated. (A, B) Subcutaneous tumors from B16F10 xenograft Balb/c nude mice were excised and photographs were taken at the termination of the experiment (A) and tumor weights were measured (B). Tumor inhibition rate (%, TIR), 67.5%. (C, D) Subcutaneous tumors from B16F10 xenograft C57BL/6 mice were excised and photopraphs were taken at the termination of the experiment (C) and xenograft tumor weights were measured (D). TIR, 80.7%. Data represents the mean±SD of tumor weights of each group (n=6). (E) FACS of CD8^+^ in CD3^+^ and GZMB^+^CD8^+^ in CD8^+^ TILs from B16F10 xenografts. (F) Representative images of IF staining of eEF2K, p-GSK3β/S9 and PD-L1 of sheEF2K and Ctrl B16F10 xenografts. (G–K) ShNT and sheEF2K-transfected B16F10 cells were injected subcutaneously into 6-week-old male C57BL/6 mice, and received CD8α mAb treatment or IgG isotype control. (G) A schematic view of the treatment plan. (H) Photopraphs of mice tumors of each group at the end of the experiment. (I) Curves of tumor growth. (J) Plots for tumor weight. (K) FACS of CD8^+^ in CD3^+^ TILs from B16F10 xenografts. ∗P<0.05; ∗∗p<0.01; ∗∗∗p<0.001. eEF2K, eukaryotic elongation factor 2 kinase; GSK3β, glycogen synthase kinase 3 beta; IF, immunofluorescence; PD-L1, programmed death ligand-1; mAb, monoclonal antibody; TIL, tumor-infiltrating lymphocyte.

The regulatory role of eEF2K in PD-L1 expression suggests that eEF2K is involved in modulating immune escape in tumor cells. To show the effect of eEF2K on tumor development and immune microenvironment, B16F10 cells with or without eEF2K depletion were orthotopically injected into the syngeneic nude mice or immunocompetent C57BL/6 mice, and the tumor volume in each mouse was measured every other day. The results showed that eEF2K knockdown significantly decreased the tumor size and weight, and this inhibitory effect was stronger in immunocompetent mice than in immunodeficient mice (figure 6A–D, online supplemental figures S4 and S5A), implying that the smaller size of tumors with eEF2K knockdown was probably due to the enhanced activity of immune cells in the tumor microenvironment. The activation of the PD-L1/PD-1 axis limits CD8^+^ T cell expansion and suppresses its antitumor activity.^38^ Consistent with this deduction, tumor-infiltrating lymphocytes were analyzed by flow cytometry, and the CD8^+^ T cell population was increased in tumors with eEF2K depletion. Also, the percentage of GZMB in CD8^+^ cells were significantly increased in tumors with eEF2K depletion (figure 6E and online supplemental figures S5B). IF staining of the tumor tissues demonstrated that the expressions of PD-L1 and phospho-GSK3β/S9 were decreased when eEF2K was knocked down (figure 6F), supporting that eEF2K modulated T cell activity by regulating PD-L1 expression in mouse model.

**Figure 7:**
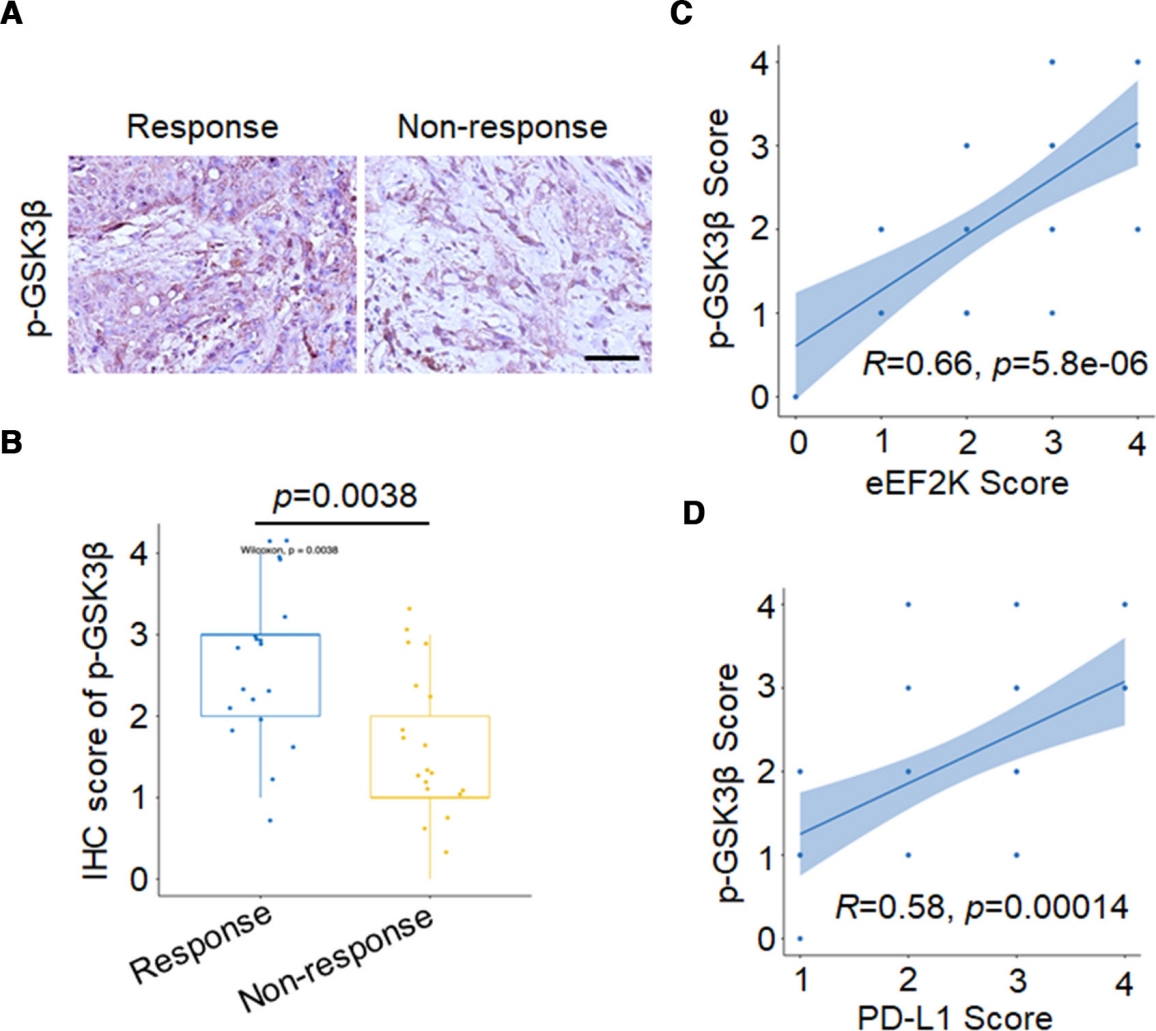
High p-GSK3β/S9 expression is positively correlated with response to anti-PD-1 immunotherapy, eEF2K and PD-L1 levels in samples from patients with melanoma. Representative images (A) and quantitative expression (B) of immunohistochemistry staining of p-GSK3β/S9 in patients with melanoma with or without response to PD-1 mAb therapy. The positive correlation between p-GSK3β/S9 and eEF2K (C), PD-L1 (D) in human melanoma specimens. eEF2K, eukaryotic elongation factor 2 kinase; GSK3β, glycogen synthase kinase 3 beta; IHC, immunohistochemical; mAb, monoclonal antibody; PD-1, programmed cell death protein-1; PD-L1, programmed death ligand-1.

To further confirm that eEF2K modulate tumor growth through the GSK3β-PD-L1 axis-regulated T cell activity, CD8α mAb was used for in vivo experiments. We observed that CD8α mAb treatment significantly enhanced tumor burden by depletion of CD8^+^ T cells in tumor (figure 6G–K, online supplemental figure S5C, D). We then validated our findings in human melanoma samples through assessing protein expression level of p-GSK3β/S9. The results showed that patients with positive response to PD-1 mAb treatment have higher p-GSK3β/S9 IHC staining scores (figure 7A, B). In particular, p-GSK3β/S9 level was positively correlated with eEF2K and PD-L1 levels in these melanoma samples (figure 7C, D), which were consistent with our in vitro and in vivo studies.

### eEF2K inhibition synergistically enhanced the therapeutic efficacy of PD-1 blockade in vivo

**Figure 8:**
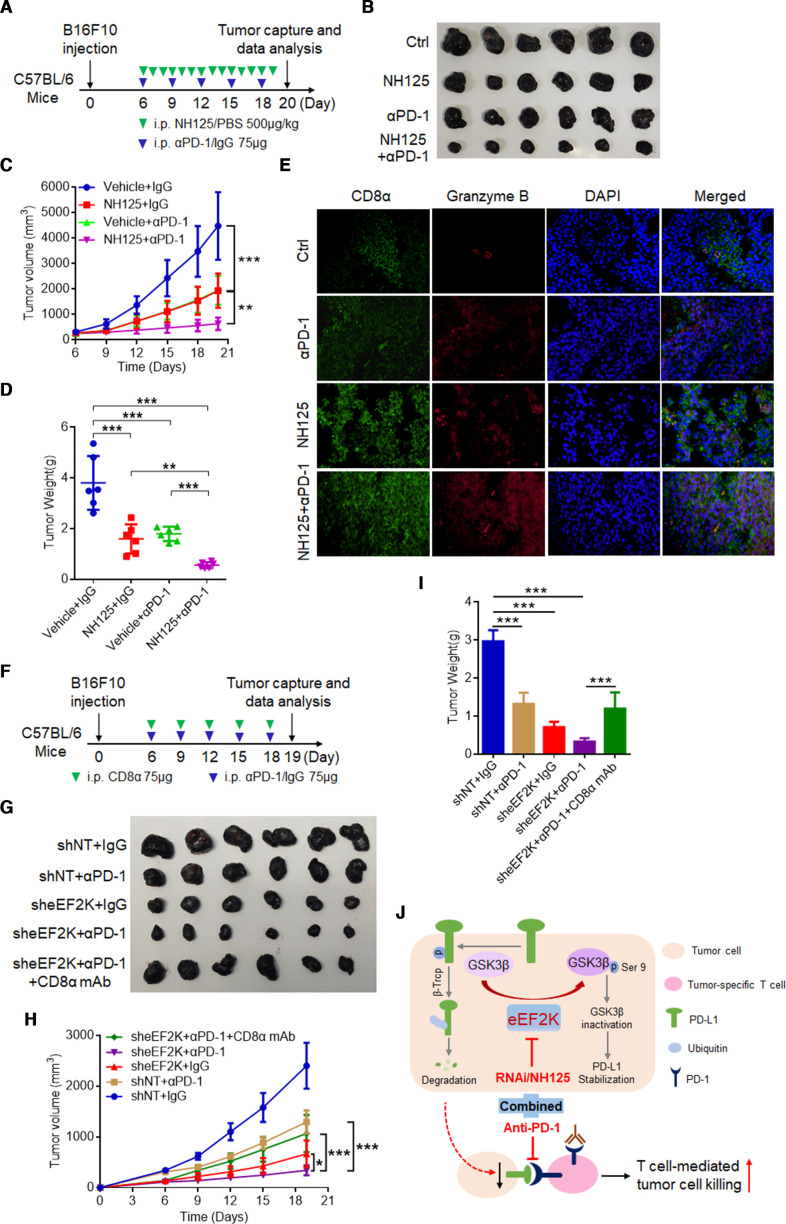
eEF2K inhibition synergistically enhanced the therapeutic efficacy of PD-1 blockade in vivo. (A–E) C57BL/6 mice were implanted with B16F10 cells and co-treated with NH125 and PD-1 mAb. (A) A schematic view of the treatment protocol. (B) Photopraphs of B16F10 tumors harvested after euthanizing the mice. n=6 for each group. (C) The tumor growth of B16F10 cells in NH125 and/or anti-PD-1 antibody-treated C57BL/6 mice. (D) Plots for tumor weight. (E) CD8α and granzyme B in mouse tumor tissues of each group were determined by immunofluorescence. (F–I) C57BL/6 mice were implanted with Ctrl or sheEF2K-transfected B16F10 cells, and received PD-1, CD8α mAb treatment or IgG isotype control. (F) A schematic view of the treatment plan. (G) Photopraphs of mice tumors of each group at the end of the experiment. (H) Curves of tumor growth. (I) Plots for tumor weight. ∗∗P<0.01; ∗∗∗p<0.001. (J) A proposed model for eEF2K-induced GSK3β-phosphorylation-dependent PD-L1 stabilization and immunoregulation in melanoma. eEF2K overexpression in cancer cells phosphorylates GSK3β at serine 9 for GSK3β inactivation, leading to PD-L1 stabilization, enhanced PD-1 interaction and subsequent immunosuppressive microenvironment as a consequent. Therapeutic depletion or inhibition of eEF2K maintains GSK3β activity for phosphorylation-dependent proteasome degradation of PD-L1, thereby decreasing the cancer cells PD-L1 expression level and synergistically enhancing the therapeutic efficacy of PD-1 mAb therapy. eEF2K, eukaryotic elongation factor 2 kinase; GSK3β, glycogen synthase kinase 3 beta; mAb, monoclonal antibody; PD-1, programmed cell death protein-1; PD-L1, programmed death ligand-1.

Our clinical data showed that 26.3% of patients with high eEF2K expression had a poor prognosis (online supplemental table S1). We next tested whether eEF2K inhibition can enhance the efficacy of PD-1 mAb therapy. We used a PD-1 mAb and NH125, an eEF2K inhibitor, to treat immune-competent mice inoculated with B16F10 melanoma cells (figure 8A). In these experiments, mice inoculated subcutaneously with B16F10 cells were randomly divided into four groups subjected to different treatments on day 6. In the B16F10 syngeneic melanoma mouse model, NH125 or PD-1 mAb lonely treatment inhibited tumor growth, while co-treatment with NH125 and PD-1 mAb achieved better efficacy, as evidenced by the greater decreases in tumor volume and tumor weight (figure 8B–D). Moreover, treatment of NH125/PD-1 mAb alone or in combination did not cause significant changes in body weight (online supplemental figure S6A), suggesting a negligible toxicity of this therapeutic regimen in tumor-bearing mice. IF microscopy showed that NH125/PD-1 mAb alone or in combination increased CD8^+^ lymphocyte infiltration and GZMB secretion in tumor tissues (figure 8E). Immunoblotting analysis showed that NH125 treatment significantly suppressed the eEF2K activity, reduced the expression levels of phosphor-GSK3β/S9 and PD-L1 (online supplemental figure S6B).

To prove that the on-target effect of NH125 is responsible for efficacy of this combination, we investigated the effect of eEF2K knockdown on PD-1 mAb therapy. We used a PD-1 mAb to treat immune-competent mice inoculated with sheEF2K melanoma cells or CTRL (figure 8F). Similarly, co-treatment with PD-1 mAb and sheEF2K further decreased the tumor volume and tumor weight compared with sheEF2K or PD-1 mAb alone treatment, and CD8α blockade rescued the decreased tumor growth induced by this combination (figure 8G–I, online supplemental figure S6C). Therefore, our results suggested that CD8^+^ T cells were required for the observed synergistic effect of combination treatment of PD-1 mAb and eEF2K inhibition. Together, these in vivo results support that eEF2K deficiency enhances the therapeutic efficacy of PD-1 mAb therapy, and may provide an effective combination therapeutic strategy for treatment of melanoma.

## Discussion

In this study, we demonstrated that eEF2K is positively associated with PD-L1 level, and that inhibition of eEF2K downregulates PD-L1 expression in melanoma. A recent published work by Proud’s group showed that eEF2K can promote PD-L1 expression in prostate and lung cancer cells.^39^ Emerging data suggest that patients whose tumors overexpress PD-L1 have better clinical outcomes with anti-PD-1 therapy. For instance, patients with PD-L1 overexpressing melanoma have a 44%–51% response rate to anti-PD-1-directed therapy, while patients with PD-L1 overexpressing non-small cell lung cancer have a 67%–100% response rate.^40^ Recent studies have reported that objective response is observed in patients with relapsed or refractory Hodgkin’s lymphoma with PD-L1 amplification in their lymphoma cells treated with anti-PD-1, whereas most progressing patients show a lack of PD-L1 upregulation in tumor cells,^41 42^ suggesting the tumor PD-L1 expression level is correlated with clinical responses to anti-PD therapy. By assessing the clinical significance of the eEF2K-PD-L1 pathway in PD-1 blockade-treated patients with melanoma, we found that high eEF2K expression could predict a better therapeutic outcome and longer OS time, suggesting eEF2K may be a potential biomarker for the efficacy of PD-1 mAb therapy.

The regulation of PD-L1 expression may affect the therapeutic effect of immune checkpoint inhibitors. For example, Li *et al* identified EGF signaling induces PD-L1 glycosylation, and inhibition of EGF-mediated PD-L1 stabilization by gefitinib sensitizes PD-1 blockade therapy in breast cancer mouse model.^11^ Another work by Ye *et al* demonstrated that MMP2/9 increases the expression level of PD-L1, and reducing PD-L1 expression by MMP2/9 inhibitor SB-3CT, enhances the therapeutic efficacy of PD-1 blockade in melanoma and Lewis lung carcinoma.^43^ Our clinical data showed that 26.3% of patients with high eEF2K expression had a poor prognosis. We demonstrated that inhibition of eEF2K decreased PD-L1, increased cytotoxic effect of CD8^+^ T cells in tumor tissues, enhanced the efficacy of PD-1 mAb treatment. These observations provide a potential effective combination therapeutic strategy. eEF2K has been found to be overexpressed in various cancers and promotes tumor progression via multiple mechanisms. Moreover, inhibition of eEF2K enhances the effects of a variety of anticancer therapies. While the current studies of eEF2K mainly focused on tumor itself, our results reveal the role of eEF2K in the regulation of tumor microenvironment, especially immune activity. eEF2K expression could predict clinical response rate to anti-PD-1/PD-L1 therapy in patients with cancer, and combinational treatment of eEF2K inhibitors can enhance the efficacy of PD-1/PD-L1 blockade and increase the tumor-infiltrating cytotoxic CD8^+^ T cell population in the tumor tissues, providing a strong rationale to develop a novel combination therapeutic strategy. Our eEF2K knockdown tumor model also confirmed the enhanced efficacy of PD-1 mAb therapy in vivo, and co-treatment with CD8 T cell elimination antibody weakened this antitumor effect, suggesting that CD8^+^ T cells contributed to the antitumor responses of the combination therapy of eEF2K inhibition with PD-1 blockade.

In this study, the immunoprecipitation-mass spectrometry analysis was conducted to identify the proteins that interact with eEF2K, and the results from this screen showed that GSK3β binds to eEF2K. GSK3β is a serine/threonine kinase that was originally considered to regulate glycogen synthase activity and glycogen synthesis.^44^ Accumulating studies have uncovered that GSK3β is an important component of Wnt/catenin signaling pathway and is involved in the regulation of tumorigenesis, invasion and metastasis.^37 45^ GSK3β-dependent substrate phosphorylation facilitates ubiquitin E3 ligase recognition and towards their ubiquitination-mediated degradation. For example, GSK3β interacts with and phosphorylates PD-L1, incorporating β-TrCP for proteasome degradation of PD-L1.^11^ Therefore, GSK3β inhibition allows PD-L1 stabilization thereby suppressing T-cell activity. In this study, we demonstrated that eEF2K directly phosphorylates GSK3β at the residue S9, contributing to GSK3β inactivation. Given that eEF2 is the only recognized substrate of eEF2K so far, our study indicates GSK3β may be a new potential substrate for eEF2K and mediates the cellular function of eEF2K. The phosphorylation and inactivation of GSK3β by eEF2K prompted us to test a hypothesis that GSK3β may mediate the effect of eEF2K on PD-L1 expression. In support of our hypothesis, the further experimental results confirmed that eEF2K-mediated GSK3β inactivation truly promotes PD-L1 stabilization and overexpression. Together with the previous study showing that eEF2K enhances PD-L1 expression in prostate and lung cancer cells via promoting PD-L1 mRNA translation and protein synthesis,^39^ the regulatory effects of eEF2K on PD-L1 may result from translational as well as post-translational modifications. It is known that dysregulation of translation elongation can cause changes in the downstream biological effects, including mRNA stability, protein expression, protein subcellular localization and co-translational protein folding. The effect of translation elongation on PD-L1 protein stability is worth to be further investigated. In tumor samples from patients with melanoma, we identified that eEF2K is positively correlated with PD-L1 and phospho-GSK3β (S9) expression. Therefore, our studies revealed molecular mechanisms underlying tumor PD-L1 regulation and uncovered an inhibitory role of eEF2K in antitumor immunity.

In summary, this study demonstrates that eEF2K enhances PD-L1 stability and expression via GSK3β inactivation, leading to cancer cells escape from immune surveillance. Therapeutic targeting of eEF2K by specific inhibitor or RNA interference results in reduced PD-L1 expression, enhanced CD8^+^ T cell-mediated tumor cell killing and synergistically boosts the effects of PD-1 blockade (figure 8J), thus making eEF2K an attractive target in designing antitumor therapies against melanoma.

## Data Availability

Data are available on reasonable request.
