# Supplementary material for: eEF2K promotes PD-L1 stabilization through inactivating GSK3β in melanoma
Source: J Immunother Cancer. 2022 Mar 28;10(3):e004026. doi: 10.1136/jitc-2021-004026 (PMC8961175; doi:10.1136/jitc-2021-004026)
Supplement: Supplementary data [file jitc-2021-004026supp001.pdf]

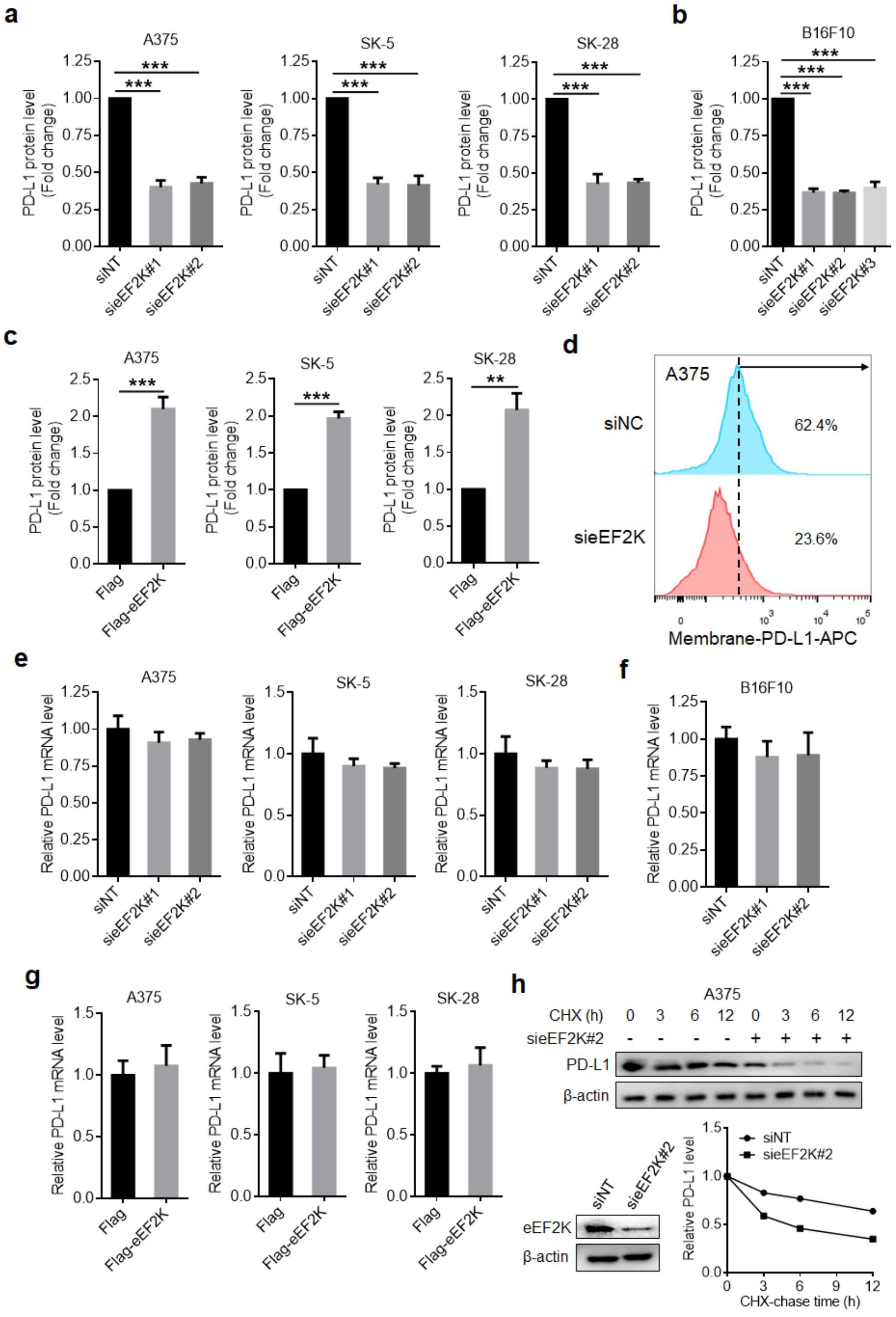

Supplementary Figure 1. eEF2K up-regulates PD-L1 expression by inhibiting its proteasome-mediated degradation. (a-c) Quantitative analysis of PD-L1 protein level. (d) FACs analysis for cell surface PD-L1 expression. (e-g) qRT-PCR analysis of

the effects of eEF2K knockdown or overexpression on PD-L1 mRNA expression. (h) Half-life analysis of PD-L1 in A375 cells transfected with siNT or sieEF2K#2. **, *P* <0.01; ***, *P* <0.001.

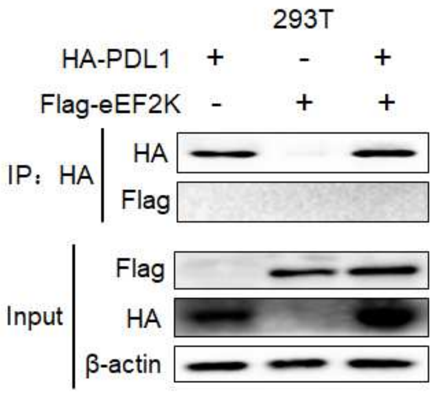

Supplementary Figure 2. **eEF2K does not interact with PD-L1.** HEK293T cells were transfected with HA-PD-L1 and Flag-eEF2K plasmids. Immunoprecipitation analysis with an anti-HA antibody was performed, and then blotted with anti-Flag antibody.

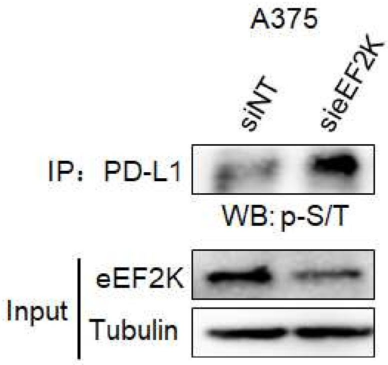

Supplementary Figure 3. eEF2K knockdown increases PD-L1 phosphorylation levels. A375 cells were transfected with a non-targeting siRNA or eEF2K-targeted

siRNAs. Immunoprecipitation analysis with an anti-PD-L1 antibody was performed, and p-S/T antibody was utilized to detect PD-L1 phosphorylation.

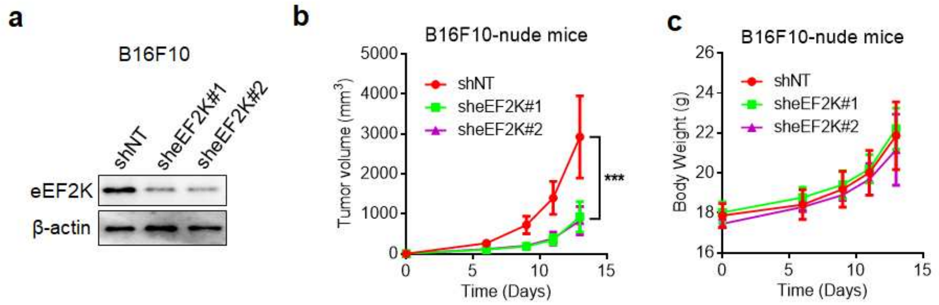

Supplementary Figure 4. Knockdown of eEF2K suppresses tumor growth in B16F10 xenograft nude mouse model. (a) eEF2K knockdown efficacy in B16F10 cells was measured by immunoblotting. (b) Tumor sizes were measured on the days as indicated. Data represents the mean ± SD of tumor sizes of each group (n = 6). ***, P <0.001. (c) Body weighs were measured on the days as indicated. Data represents the mean ± SD of body weighs of each group (n = 6).

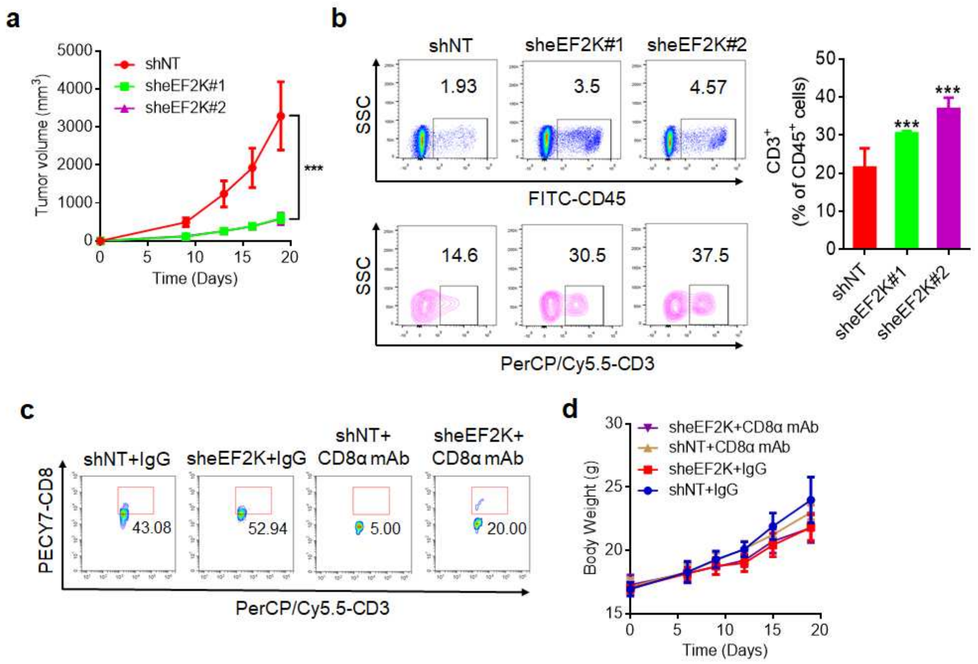

Supplementary Figure 5. Knockdown of eEF2K promotes T cell activity in B16F10 xenograft tumor. (a) The sizes of xenograft tumors from C57BL/6 mice were measured on the days as indicated. Data represents the mean ± SD of tumor sizes of each group (n = 6). ***, *P* <0.001. (b) FACS of CD3+ in CD45+ cells from B16F10 xenografts. ***, *P* <0.001. (c) FACS of CD8+ in CD3+ cells from B16F10 xenografts. (d) Plots for body weight for the indicated treatment.

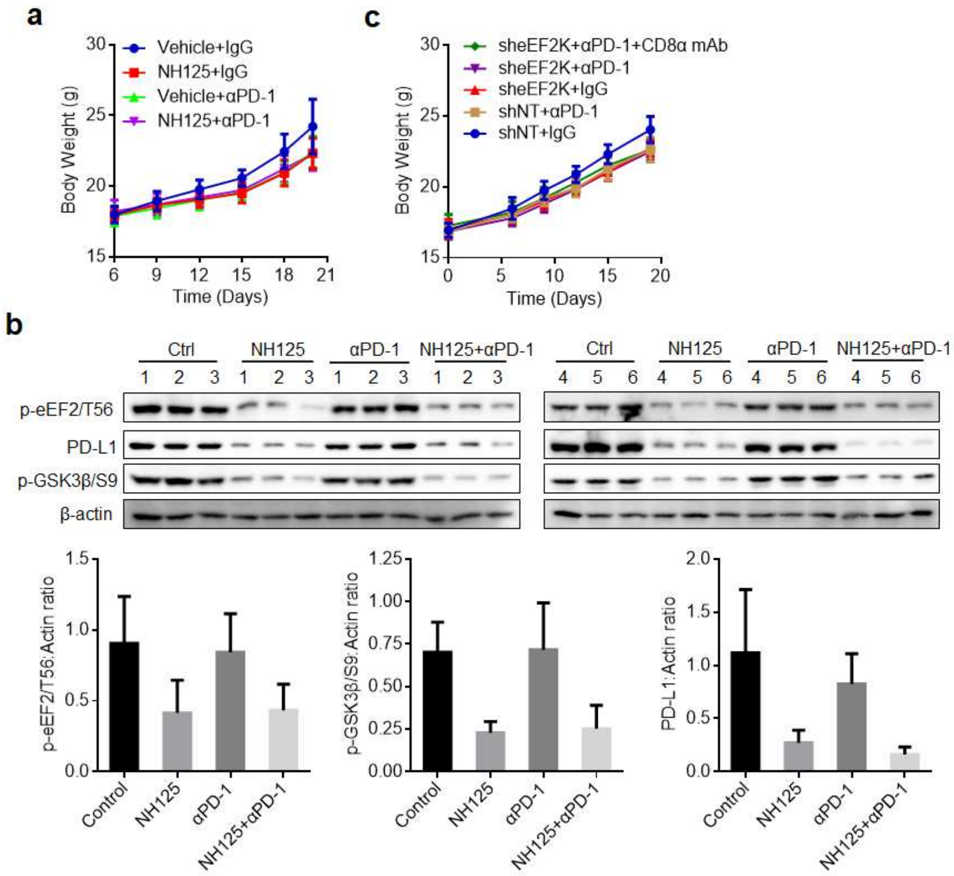

Supplementary Figure 6. eEF2K inhibition synergistically enhanced the therapeutic efficacy of PD-1 blockade in vivo. (a,c) Plots for body weight for the indicated treatment. (b) p-eEF2/T56, PD-L1 and p-GSK3β/S9 expressions in the tumor tissues were detected and quantified.

Table S1. The correlation analysis between eEF2K expression and prognosis

	Prognosis		
	Good	Poor	Total
eEF2K expression			
Low	5 (13.2%)	4 (10.5%)	9
High	19 (50%)	10 (26.3%)	29
Total	24	14	38
